# An extinct vertebrate preserved by its living hybridogenetic descendant

**DOI:** 10.1038/s41598-017-12942-y

**Published:** 2017-10-06

**Authors:** Sylvain Dubey, Christophe Dufresnes

**Affiliations:** 1 Department of Ecology & Evolution, University of Lausanne, Biophore Building, 1015 Lausanne, Switzerland; 2 Hintermann & Weber SA, Rue de l’Eglise-Catholique 9b, 1820 Montreux, Switzerland; 3 Department of Animal & Plant Sciences, University of Sheffield, Alfred Denny Building, Western Bank, Sheffield, S10 2TN United Kingdom

## Abstract

Hybridogenesis is a special mode of hybrid reproduction where one parental genome is eliminated and the other is transmitted clonally. We propose that this mechanism can perpetuate the genome of extinct species, based on new genetic data from *Pelophylax* water frogs. We characterized the genetic makeup of Italian hybridogenetic hybrids (*P*. kl. *hispanicus* and *esculentus*) and identified a new endemic lineage of Eastern-Mediterranean origin as one parental ancestor of *P*. kl. *hispanicus*. This taxon is nowadays extinct in the wild but its germline subsists through its hybridogenetic descendant, which can thus be considered as a “semi living fossil”. Such rare situation calls for realistic efforts of de-extinction through selective breeding without genetic engineering, and fuels the topical controversy of reviving long extinct species. “Ghost” species hidden by taxa of hybrid origin may be more frequent than suspected in vertebrate groups that experienced a strong history of hybridization and semi-sexual reproduction.

## Introduction

**Figure 1:**
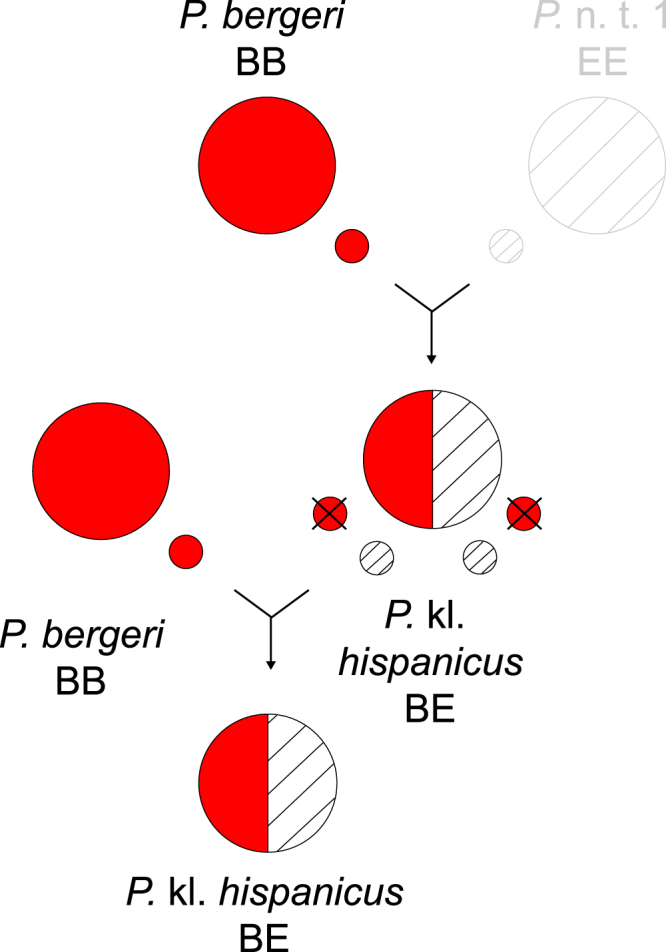
Hybridogenesis in the *P*. kl. *hispanicus* complex. *Pelophylax* kl. *hispanicus* originates from hybridization between *P. bergeri* (B genome) and a new lineage *P*. n. t. 1 (E genome, as a reference to “Extinct”). It eliminates its *P. bergeri* germline and thus only contributes *P*. n. t. 1 gametes. This klepton requires *P. bergeri* to reproduce and their crosses yield klepton offspring only. These natural mechanisms allow to preserve the *P*. n. t. 1 germline in the wild.

In a context of the worldwide mass extinction of biodiversity, the hopes for rediscovering or reviving extinct wildlife have become a popular scientific fantasy^1,2^. So-called “de-extinction” programs, like the “mammophant” (mammoth × elephant *in vitro* hybrid) or the Lazarus project (cloning of the extinct platypus frog from frozen specimens) are taking this fantasy to the experimental level, following recent advances in cloning biotechnology^3–7^. Here we present a new opportunity: the preservation of the genome of an extinct species through hybridogenesis, a special reproductive mode of hybrids. In this mode, the parental genomes do not recombine in F1s: one is eliminated before meiosis, and the other is solely transmitted in a clonal manner^8^ (Fig. 1). Such case of semi-sexual reproduction is rare, but found in several amphibians and fishes^9^. It is particularly famous of European water frogs, where pool frogs (*P. lessonae* sensu lato) hybridize with marsh frogs (*P. ridibundus* s. l.) to form hybridogenetic hybrid species, aka kleptons, (*P*. kl. *esculentus* s. l.), in which the *P. lessonae* s. l. genome is excluded from the germline^8^. This group thus makes a fascinating model system to study hybridogenesis, where it can have far-reaching consequences: invasive marsh frogs are currently replacing pool frogs across Western Europe^10^; human-mediated introductions have led to the formation of novel hybridogenetic systems from naturally allopatric taxa, involving new klepton species^11^. As such, and while they benefit from a specific status, kleptons usually have a poor conservation value as they act as genetic parasites. In this report, we propose that hybridogenesis has perpetuated the lineage of an extinct clade of water frogs in Italy, and that it could be regarded as a potential mean to resurrect such extinct species.

**Figure 2:**
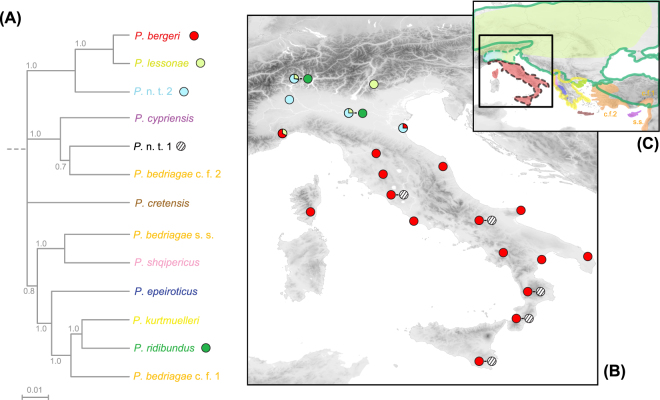
(**A**) Nuclear phylogeny of Western Palearctic water frogs based on two nuclear loci combined (~1,600 bp, SAI-1 + CMI-2). Branch support are shown for internal nodes. (**B**) Distribution of Italian lineages from our sampling. (**C**) Distribution of water frog species, according to IUCN red list and our study (dash line: extinct range in Italy). *Pelophylax* c. f. *bedriagae* is a species complex involving multiple cryptic taxa summarized as *P. bedriagae* s. s., cf. 1 and cf. 2, based on SAI-1 and mtDNA variation^18^. For a mitochondrial phylogeny, see refs^11,16^. For information on the reference sequences, see Table S1 and references therein. Nearby Swiss Ticino localities were pooled for visibility. The map was built using ArcGIS 9.3 (http://www.esri.com/arcgis/about-arcgis).

The Apennine Peninsula (Italy and Swiss Ticino) is widely inhabited by pool frogs and their associated kleptons: *P. lessonae* with *P*. kl. *esculentus* north of the Apennine Mountains and its sister species *P. bergeri* with *P*. kl. *hispanicus* in the Apennines and in Sicily^12^ (Fig. 2). Yet, marsh frogs have never been reported over the Peninsula^12,13^, suggesting that either they did not contribute to its colonization, or they became extinct since. Their origin and nature have thus remained a mystery. By parsing nuclear sequence data from two independent intronic regions (abbreviated SAI-1 and CMI-2) together with microsatellite data, we characterized these ancestral marsh frog germlines in a comprehensive phylogeographic framework including most *Pelophylax* taxa known from the Western Palearctic. We made three striking discoveries.

First, we identified the marsh frog ancestor of *P*. kl. *hispanicus* as a new monophyletic lineage that we refer to as *Pelophylax* n. t. 1 (Fig. 2). This “ghost” species, widespread south of the Apennines, is supported by the SAI-1 phylogeny, which distinguishes all Western-Palearctic species (Fig. S1), and is represented by specific haplotypes for the shorter CMI-2 (Fig. S2). Some of the latter were also sampled north of the Apennines, as a probable result of past hybridization or incomplete lineage sorting among marsh frogs for this less polymorphic marker (Fig. S2; as also illustrated by unresolved *P. ridibundus*/*P. cypriensis* alleles). Potential signs of ancient admixture also arise from the microsatellite data: *P*. n. t. 1 is distinguished from the North-Italian marsh frog germline (see below), but few individuals have intermediate clustering (Fig. S3). Phylogenetic analyses show that *P*. n. t. 1 was most closely related to Eastern-Mediterranean water frogs, namely the Cyprian-endemic *P. cypriensis*, as well as Western Anatolian lineages of the *P*. c.f. *bedriagae* complex (Fig. 2), from which it diverged during the Pleistocene (1.5 Mya, 95% HPD 0.6-2.6; Fig. S4). This timing parallels the split between the pool frogs *P. lessonae* and *P. bergeri* north and south of the Apennines during the Calabrian (estimated at ~1.3 Mya^14^), a Pleistocene period characterized by extreme cold and desiccation. Nowadays, *P*. n. t. 1 appears to be extinct as such, and its genome only persists in the hybridogenetic hybrid *P*. kl. *hispanicus* (Fig. 1).

Second, we identified the marsh frog ancestor of kleptons *P*. kl. *esculentus* from N-Italy/S-Switzerland as the European marsh frog *P. ridibundus*, that currently inhabits most of the Western Palearctic^12,13^ (Fig. 2, Fig. S1, Fig. S2). The presence of different marsh frog lineages south and north of the Apennines is in clear accordance with well-established biogeographic paradigms, notably for amphibians, and especially pool frogs^14^.

Third, we unexpectedly found a new cryptic clade of pool frogs restricted to NW-Italy and S-Switzerland (Ticino; Fig. 2, Fig. S1, Fig. S2), diverged as early as the Late-Pliocene (3.0 Mya, 95% HPD 1.2-4.9) that we refer to as *Pelophylax* n. t. 2. It appears deeply admixed and panmictic with *P. lessonae*, as also suggested by the microsatellites (Fig. S3). This lineage might stem from long term isolation in the remote Alpine valleys (as in other herps^15^), before secondary contact and introgression by *P. lessonae*. Alternatively, it could be of hybrid origin resulting from leaky hybridogenesis between pool and marsh frogs, which would explain the intermediate phylogenetic position (Fig. 2), together with the absence of reciprocal mitochondrial divergence^16^.

Although our sequence markers discriminate all known species^17,18^, the current data is insufficient to accurately reconstruct the biogeography of marsh frogs of the Apennine Peninsula. Notably, it is unclear whether *Pelophylax*. n. t. 1 have speciated in the Italian Peninsula or colonized it from Central/Eastern Europe, where it would have become extinct as well. It is however unlikely that the divergences took place solely in the kleptons after hybridogenesis was initiated: *P*. n. t. 1 would otherwise cluster as a sister species of *P. ridibundus* found north of the Apennines, which we can reject from the phylogeny (Fig. 2). The contrasted genetic structures between *P. bergeri* and *P*. kl. *hispanicus* (and their marsh frog germlines) also argue for an independent evolution of *P*. n. t. 1 (Fig. S3). However, clonality may have boosted the evolutionary rate of this lineage and overestimated our dating estimates. In addition, the intronic markers used could yield biased phylogenetic signals due to specific features, like retrotransposons^17^. Given the complex evolutionary history of Western Palearctic water frogs, involving frequent events of hybridization between species^19,20^, robust nuclear phylogenies and admixture analyses will require high-throughput genomic data.

If it was ever present in Italy, why this taxon disappeared remains a mystery. Water frogs fossils from the middle Pleistocene to the Holocene are found throughout Italy^21^ but distinguishing between potential *Pelophylax* residents is challenging with current biometric methods^22^. Yet, many of these records were attributed to marsh frogs “*P. ridibundus*”^23^ and could thus belong to *P*. n. t. 1.

The persistence of the genetic legacy of an extinct taxon provides exciting insights. By carrying this lineage, *P*. kl. *hispanicus* can thus be considered as a “semi-living fossil” and deserves a strong conservation value. The same is true for *P. bergeri* on which *P*. kl. *hispanicus* relies for reproduction. Beyond its fascinating mechanism of genetic preservation, this system fuels the fantasy for de-extinction. Thanks to the hybridogenetic context, we envisage simple ways that would allow to resuscitate *P*. n. t. 1 without genetic engineering. Theoretically, crossings between *P*. kl. *hispanicus* individuals would produce pure *P*. n. t. 1 offspring. However, most of these crossings are sterile, otherwise marsh frogs would be naturally found in Central and South-Italy. Like in other water frog hybridogenetic systems, the *P*. n. t. 1 germline likely degenerated following many generations of clonal transmission (ref.^24^ and references therein). However, intense efforts of multiple controlled crossing experiments may allow to obtain few viable offspring, i.e. those where different deleterious mutations were fixed in their parental germlines. Although audacious, these might be rewarding given the flexibility of amphibian development; e.g. eggs can sometimes still develop under artificial haploid parthenogenesis (notably in *Pelophylax*^25^). An alternative approach would be to cross *P*. kl. *hispanicus* with its closest living relatives, e.g. the Anatolian lineage *P. bedriagae* c. f. 2. Subsequent backcrossing of the resulting F1 *P*. n. t. 1 × *P*. c. f. *bedriagae* hybrids with *P*. kl. *hispanicus* might allow to dilute the *P*. c. f. *bedriagae* genome while simultaneously purging deleterious mutations accumulated on the *P*. n. t. 1 germline. Selective breeding is a common de-extinction practice, e.g. envisaged to resurrect the aurochs^26^ and applied to recreate the quagga (an extinct subspecies of zebra), although the resulting individuals could not be genetically identical^27^. The implementation of such approaches would contribute to the ethical debate of reviving long extinct species. De-extinction is questionable when it pursues a technological achievement^28^. While this would not be the case here, the responsibility of mankind in restoring a biodiversity that “naturally” vanished prior to the sixth mass extinction still remains a topic of extreme controversy^28^. More generally, the de-extinction possibilities offered by scientific advances should not defuse the dramatic measures required to protect wildlife on the verge of extinction.

This study calls for the tracking of “ghost” vertebrate lineages that remained hidden and protected in other species of hybrid origins, through hybridogenesis or other mechanisms with potentially similar consequences (e.g. allopolyploidisation^29,30^). Unknown lineages underlie the genetic makeup of some polyploid plants^31^ and fishes^32^. Such taxa may be more frequent than imagined in vertebrates, especially in amphibians and fishes where these reproductive modes have been well-documented^9,33^.

## Methods

### DNA Sampling

We included 77 individuals sampled across Italy and S-Switzerland (Ticino), representing 23 localities (Table S1). Eleven samples from the different Western-Palearctic species of water frogs were also included for sequencing, to complement available data (see below). DNA was obtained from non-invasive buccal swabs or ethanol-preserved tissues, and was extracted with the Qiagen Blood & Tissue DNA extraction kit (Qiagen, Netherlands). Procedures were approved by the local and national ethics committees for animal experiments (karch Ticino) and performed in accordance with their guidelines and regulations. The mitochondrial lineage of all but three individuals was available from^16^.

### Microsatellite analyses

Given the uncertainty to identify water frogs by morphology^16^, individuals from the study area were genotyped at microsatellite loci with diagnostic alleles to distinguish between pool (here *P. bergeri* /*lessonae*) from marsh frogs (*P. ridibundus* s. l.) frogs, as well as their kleptons (here *P*. kl. *esculentus*/*hispanicus*)^10,16,34^. This also confirmed the expected absence of marsh frogs (*P. ridibundus* s. l.).

To this purpose and to infer population structure, we analyzed nine polymorphic microsatellites in 72 individuals of pool and edible frogs from the study area (loci and methods^16^). Furthermore, we were able to parse the genotypes of 8 markers in 26 kleptons and reconstructed their marsh frog haplotypes, as previously done in Western Switzerland^10^. Both datasets (pool frogs/kleptons and phased marsh frog haplotypes) were analyzed by Principal Component Analyses (PCA) on individual genotypes, using the *ade4* and *adegenet* packages in R. The differences between pool frogs and their associated kleptons stem from their marsh frog germlines. The genetic structure of kleptons should thus mirror the genetic structure of pool frogs if their marsh frog germlines only co-evolved in the kleptons after hybridogenesis was initiated, but not necessarily if the divergence predate hybridogenesis.

### Sequencing of nuclear introns

We amplified and sequenced intronic portions of the nuclear genes *Serum albumin* (intron 1, abbreviated SAI-1) and *Cellular myelocytomatosis* (intron 2, abbreviated CMI-2) in respectively 45 (34 pool frogs and 11 kleptons) and 60 (49 pool frogs and 11 kleptons) individuals from the study area. These two markers were chosen as they have been widely used for phylogeography of water frogs and were consistently shown to discriminate between currently recognized taxa^16,18,35^. PCR conditions and primers are available in Table S2. We first attempted direct sequencing and then cloned heterozygous individuals. For SAI-1, we developed a set of primers specific to *P. bergeri*/*lessonae* alleles and a set of primers specific to the alleles of other taxa (Fig. S5). This allowed to independently amplify and sequence the two alleles of kleptons that harbored a *P. bergeri* or a *P. lessonae* allele (i.e. *P. bergeri*/*P*. n. t. 1 and *P. lessonae*/*P. ridibundus*). However, kleptons with a *P*. n. t. 2 allele, as well as heterozygous pool frogs had to be cloned. Cloning was performed with the TOPO-cloning kit (Invitrogen), and a minimum of eight clones per individual was sequenced.

### Phylogenetic analyses

We complemented our new data with published sequences available for these two genes. For SAI-1, this included the haplotypes found across the ranges of all species^16–18^ (see Table S1 for details). For CMI-2, this included haplotypes from *P. lessonae*, *P. bergeri* and a *P. ridibundus* × *P. bedriagae* c. f. 1 hybrid from E-Turkey^35^. GenBank accession numbers of haplotypes analyzed in this study are provided in Table S3. Sequences were manually aligned in Seaview^36^. Phylogenetic analyses were performed on 59 SAI-1 and 31 CMI-2 haplotypes from 15 taxa (including *P*. n. t. 1 and *P*. n. t. 2), with the Asian *P. nigromaculatus* and the early-diverged European *P. perezi* as outgroups. Phylogenetic Bayesian inferences were performed with MrBayes^37^, with 10 million iterations and 2 chains. Evolutionary models were chosen according to JModelTest^38^ (SAI-1: GTR + G; CMI-2: HKY + G). We also computed haplotype networks using TCS^39^ to visualize haplotype variation, including indels. In addition to marker-specific trees, we built a phylogeny based on concatenated sequences representative of the variation of each species for the two markers.

We performed molecular dating by calibrating the phylogeny to the known split of *P. cretensis* at the end of the Messinian salinity crisis (~5.3-5.5 Mya)^40,41^. To this purpose, we estimated the mutation rates of our nuclear markers beforehand from the evolution of the mitochondrial genomes published for ten species of European and Asian water frogs, which provides a well-resolved phylogeny^42^. Analyses were conducted in BEAST using a Yule-process prior, an uncorrelated lognormal relaxed clock^43^ and different partitions for each sequence set. This calibration involved a normally distributed prior at 5.4 Mya (95% HDP 4.8-5.9) for the split of *P. cretensis*. Then, we applied the molecular clock on the nuclear dataset using the estimated mutations rates with normally-distributed priors covering their 95% HDP (SAI-1: 0.0056 (0.0027-0.0112); CMI-2: 0.0113 (0.0026-0.0286)). We ran two independent chains of 20 million iterations each (with the first 2 million excluded as burnin) and used the Tracer module to check for convergence and effective sample size of parameters. We built a time-calibrated phylogeny from the BEAST runs with the module TreeAnnotator and the R package *phyloch*^44^.

## Electronic supplementary material

Supplementary Information

## References

[1] Kumar S (2012). Extinction need not be forever. Nature..

[2] Biton R (2013). The rediscovered Hula painted frog is a living fossil. Nat. Comm..

[3] Lanza R (2000). Cloning of an endangered species (*Bos gaurus*) using interspecies nuclear transfer. Cloning..

[4] Loi P (2001). Genetic rescue of an endangered mammal by cross-species nuclear transfer using post-mortem somatic cells. Nat. Biotechnol..

[5] Clarke, S. Bizarre extinct frog brought back to life. ABCNews. http://www.abc.net.au/news/2013–03–16/bizarre-extinct-frog-brought-back-to-life/4575916 (2013).

[6] Shapiro B (2016). Mammoth 2.0: will genome engineering resurrect extinct species. Genome Biol..

[7] Reardon S (2016). Welcome to the CRISPR zoo. Nature..

[8] Graf JD, Müller WP (1979). Experimental gynogenesis provides evidence of hybridogenetic reproduction in the *Rana esculenta* complex. Experientia..

[9] Avise, J. C. Reproduction by the Semichaste: Gynogenesis, Hybridogenesis and kleptogenesis. In *Clonality: the genetics, ecology, and evolution of sexual abstinence in vertebrate animals*. (Oxford, 2008).

[10] Leuenberger J, Gander A, Schmidt BR, Perrin N (2014). Are invasive marsh frogs (*Pelophylax ridibundus*) replacing the native *P. lessonae*/*P. esculentus* hybridogenetic complex in Western Europe? Genetic evidence from a field study. Conserv. Genet..

[11] Dufresnes C, Denöel M, di Santo L, Dubey S (2017). Triple invasion of *Pelophylax* water frogs in southern France, potentially inducing a new hybridogenetic complex. Sci. Rep..

[12] Nöllert, A. & Nöllert, C. (2003). *Rana ridibunda*. In *Guide des amphibiens d’Europe* 341–346 (Delachaux et Niestlé, 2003).

[13] Kuzmin, S. *et al*. “*Pelophylax ridibundus*”. The IUCN Red List of Threatened Species. e.T58705A11825745 (2009).

[14] Canestrelli D, Nascetti G (2008). Phylogeography of the pool frog *Rana* (*Pelophylax*) *lessonae* in the Italian Peninsula and Sicily: multiple refugia, glacial expansions and nuclear-mitochondrial discordance. J. Biogeogr..

[15] Ghielmi S, Menegon M, Marsden SJ, Laddaga L, Ursenbacher S (2016). A new vertebrate for Europe: The discovery of a range-restricted relict viper in the western Italian Alps. J. Zoolog. Syst. Evol. Res..

[16] Dufresnes C (2017). Cryptic invasion of Italian pool frogs (*Pelophylax bergeri*) across Western Europe unraveled by multilocus phylogeography. Biol. Invasions..

[17] Plötner J (2009). Evolution of serum albumin intron-1 is shaped by a 5′ truncated non-long terminal repeat retro- transposon in western Palearctic water frogs (Neobatrachia). Mol. Phylogenet. Evol..

[18] Plötner J (2012). Genetic data reveal that water frogs of *Cyprus* (genus *Pelophylax*) are an endemic species of Messinian origin. Zoosyst. Evol..

[19] Herczeg D, Vörös J, Christiansen DG, Benovics M, Mikulicek P (2017). Taxonomic composition and ploidy level among European water frogs (Anura: Ranidae: *Pelophylax*) in eastern Hungary. J. Zoolog. Syst. Evol. Res..

[20] Kolenda K, Pietras-Lebioda S, Hofman M, Ogielska M, Pabijan M (2017). (2017). Preliminary genetic data suggest the occurrence of the Balkan water frog, *Pelophylax kurtmuelleri*, in southwestern Poland. Amphib-reptil..

[21] Holman, J. A. In *Pleistocene amphibians and reptiles in Britain and Europe* 63-66 (Oxford, 1998).

[22] Blain HA, Lozano-Fernandez I, Böhme G (2015). Variation in the ilium of central European water frogs *Pelophylax* (Amphibia, Ranidae) and its implications for species-level identification of fragmentary anuran fossils. Zool. Stud..

[23] Böhme, M. & Ilg, A. fosFARbase, www.wahre-staerke.com [accessed July 2017] (2003).

[24] Vorburger, C., Schmeller, D. S., Hotz, H., Guex, G. D. & Reyer, H. U. Masked damage: mutational load in hemiclonal water frogs. in *Lost sex: the evolutionary biology of parthenogenesis* 433–446 (Springer, 2009).

[25] Mittwoch UP (1972). J. Med. Genet..

[26] Sinding MS, Gilbert MTP (2016). The draft genome of extinct European aurochs and its implications for de-extinction. Open Quaternary..

[27] Harley EH, Knight MH, Lardner C, Wooding B, Gregor M (2009). The Quagga project: progress over 20 years of selective breeding. S. Afr. J. Wildl. Res..

[28] Sandler R (2013). The ethics of reviving long extinct species. Conserv. Biol..

[29] Comai L (2005). The advantages and disadvantages of being polyploid. Nat. Rev. Genet..

[30] Janko K, Bohen J, Lamatch D, Flajshans M (2007). The gynogenetic reproduction of diploid and triploid hybrid spined loaches (Cobitis: Teleostei), and their ability to establish successful clonal lineages – on the evolution of polyploidy in asexual vertebrates. Genetica..

[31] Luo X (2017). Chasing ghosts: allopolyploid origin of *Oxyria sinensis* (Polygonaceae) from its only diploid congener and an unknown ancestor. Mol. Ecol..

[32] Crespo-López ME, Pala I, Duarte TL, Dowling TE, Coelho MM (2007). Genetic structure of the diploid-polyploid fish *Squalius alburnoides* in southern Iberian basins Tejo and Guadiana, based on microsatellites. J. Fish. Biol..

[33] Schmid M, Evans BJ, Bogart JP (2015). Polyploidy in Amphibia. Cytogenet. Genome Res..

[34] Hotz H (2001). Microsatellites: a tool for evolutionary genetic studies of western Palearctic water frogs. Mitt. Mus. Naturkunde Berl. Zoolog. Reihe.

[35] Komaki S (2015). Robust molecular phylogeny and palaeodistirbution modelling resolve a complex evolutionary history: glacial cycling drove recurrent mtDNA introgression among *Pelophylax* frogs in East Asia. J. Biogeogr..

[36] Gouy M, Guindon S, Gascuel O (2010). SeaView version 4: a multiplatform graphical user interface for sequence alignment and phylogenetic tree building. Mol. Biol. Evol..

[37] Ronquist F, Huelsenbeck JP (2003). MrBayes 3: Bayesian phylogenetic inference under mixed models. Bioinformatics..

[38] Darriba D, Taboada GL, Doallo R, Posada D (2012). jModelTest 2: more models, new heuristics and parallel computing. Nat. Methods..

[39] Clement M, Posada D, Crandall KA (2000). TCS: a computer program to estimate gene genealogies. Mol. Ecol..

[40] Beerli P, Hotz H, Uzzel T (1996). Geological dated sea barriers calibrate a protein clock for Aegean water frogs. Evolution.

[41] Kriigsman W, Hilgen FJ, Raffi I, Sierro FJ, Wilson DS (2000). Chronology, causes and progression of the Messinian salinity crisis. Nature..

[42] Hofman S, Pabijan M, Osikowski A, Litvinchuk SN, Szymura JM (2016). Phylogenetic relationships among four new complete mitogenome sequences of *Pelophylax* (Amphibia: Anura) from the Balkans and Cyprus. MDN..

[43] Drummond AJ, Rambaut A (2007). BEAST: Bayesian evolutionary analysis by sampling trees. BMC Evol. Biol..

[44] Heibl, C. PHYLOCH: R language tree plotting tools and interfaces to diverse phylogenetic software packages. http://www.christophheibl.de/Rpackages.html (2013).

