# Author Correction: An extinct vertebrate preserved by its living hybridogenetic descendant

**DOI:** 10.1038/s41598-018-23429-9

**Published:** 2018-04-12

**Authors:** Sylvain Dubey, Christophe Dufresnes

**Affiliations:** 1 Department of Ecology & Evolution, University of Lausanne, Biophore Building, 1015 Lausanne, Switzerland; 2 Hintermann & Weber SA, Rue de l’Eglise-Catholique 9b, 1820 Montreux, Switzerland; 3 Department of Animal & Plant Sciences, University of Sheffield, Alfred Denny Building, Western Bank, Sheffield, S10 2TN United Kingdom

Correction to: *Scientific Reports* 10.1038/s41598-017-12942-y, published online 06 October 2017

The Supplementary Information file originally published with this Article should have two Genbank accession numbers omitted in Table 3. These errors have been corrected in the Supplementary Information that now accompanies the Article.

