# Supplementary material for: An extinct vertebrate preserved by its living hybridogenetic descendant
Source: Sci Rep. 2017 Oct 6;7:12768. doi: 10.1038/s41598-017-12942-y (PMC5630569; doi:10.1038/s41598-017-12942-y)
Supplement: Supplementary file 1 — Supplementary Information [file 41598_2017_12942_MOESM1_ESM.pdf]

Supplementary Materials for

AN EXTINCT VERTEBRATE PRESERVED BY ITS LIVING HYBRIDOGENETIC DESCENDANT

Sylvain Dubey and Christophe Dufresnes

Correspondence to c.dufresnes@sheffield.ac.uk

This PDF file includes:

Figs. S1 to S5

Tables S1 to S3

Fig. S1: **(A) Phylogenetic reconstruction and (B) haplotype network of SAI-1 (1399bp aligned).**

Branch support are shown for major branches. Haplotype were named after the taxonomic identity of samples. BED02 and BED03 correspond to *P. bedriagae* c. f. 1 alleles, probably acquired through introgressive hybridization. *Pelophylax* n. t. 1 and n. t. 2 alleles were labelled EXT and TIC, respectively, as a reference to "Extinct" and "Ticino" (where *P.* n. t. 2 was massively found).

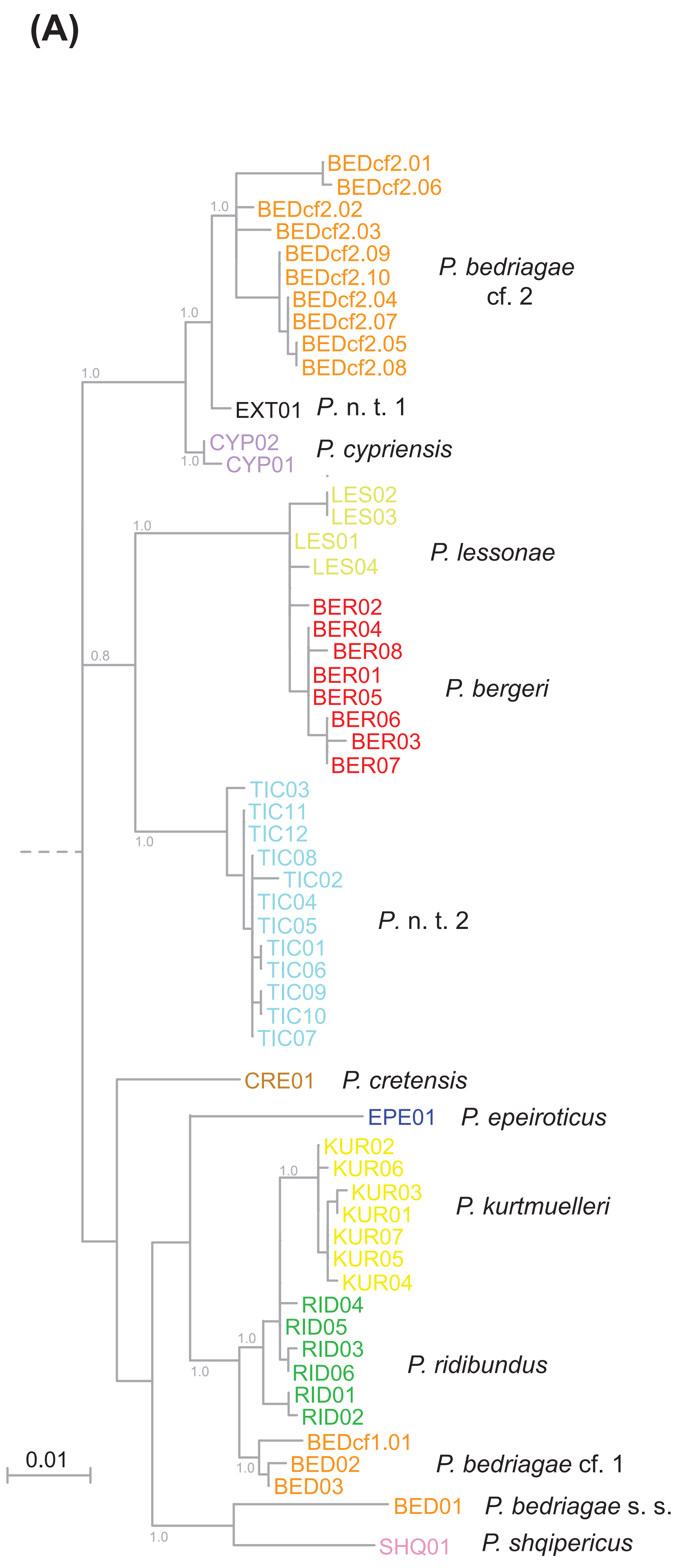

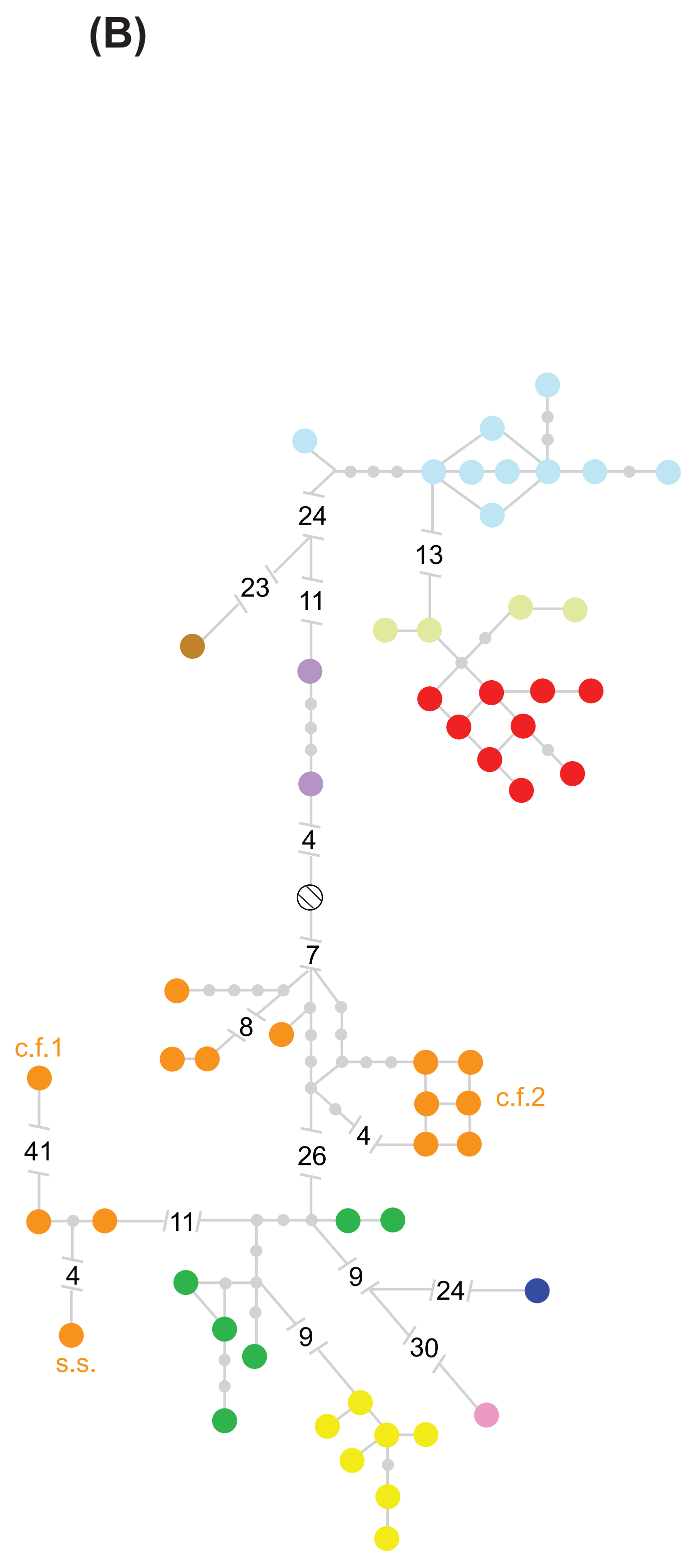

Fig. S2: **(A) Phylogenetic reconstruction and (B) haplotype network (B) of CMI-2 (232bp aligned).**

Branch support are shown for major branches. Haplotypes were named after the taxonomic identity of samples; *P. cypriensis* shows incomplete lineage sorting with *P. ridibundus* (identical RID04 and CYP01); RID05-08 were exclusively sampled in N-Italy / S-Switzerland and may represent *P.* n. t. 1 alleles present north of the Appennines, or incomplete lineage sorting between *P.* n. t. 1 and N-Italian *P. ridibundus*. *Pelophylax* n. t. 1 and n. t. 2 alleles were labelled EXT and TIC, respectively, as a reference to "Extinct" and "Ticino" (where *P.* n.t. 2 was massively found).

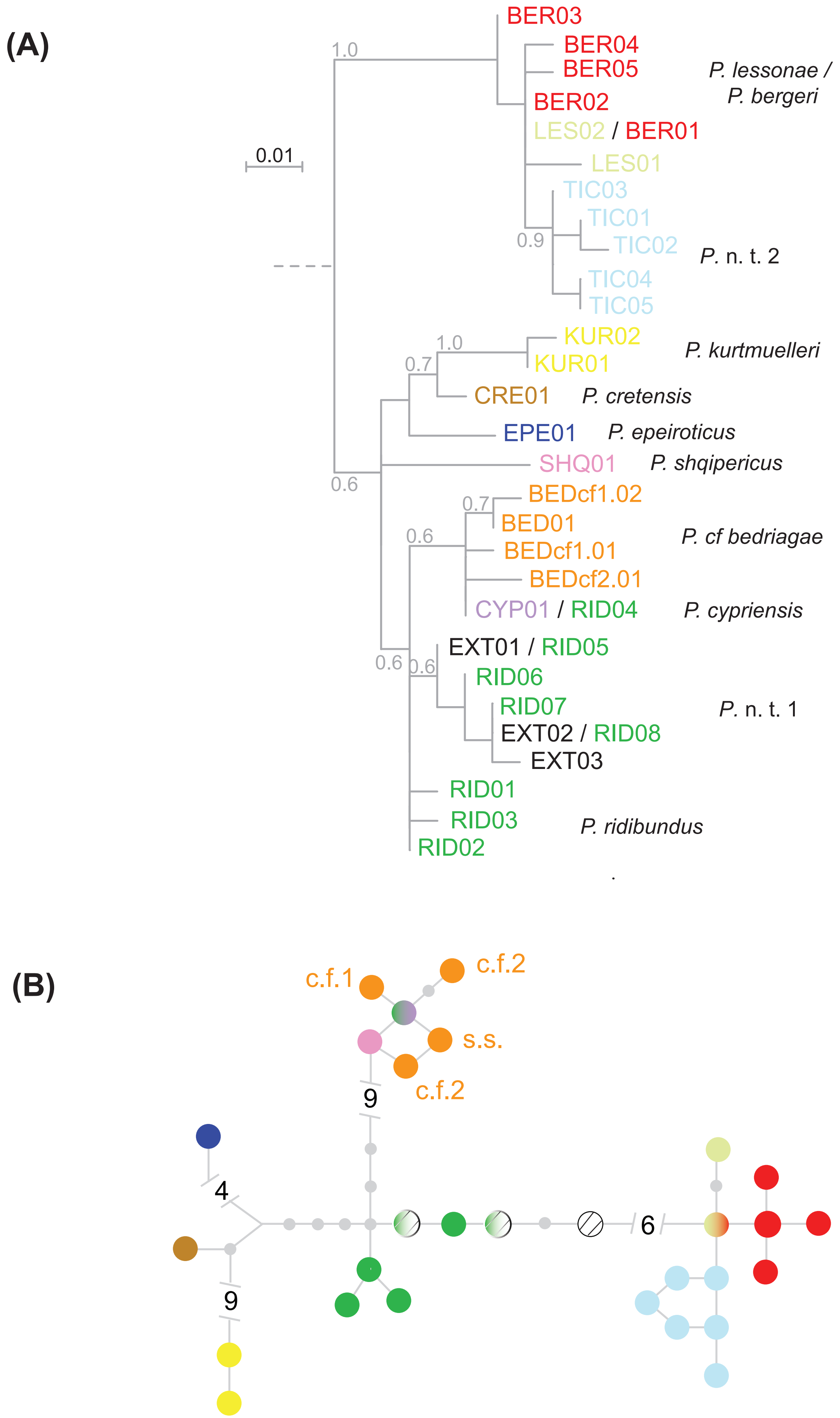

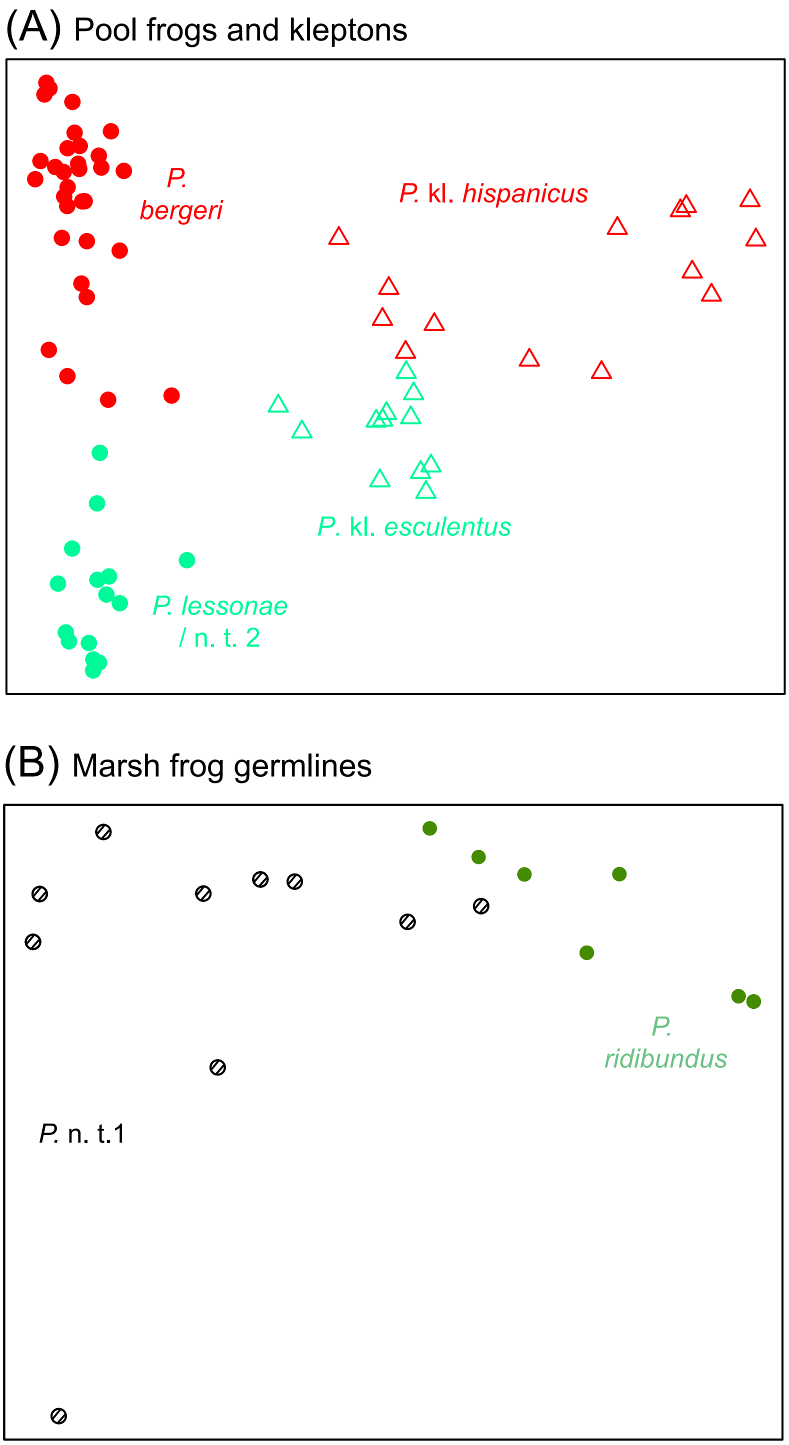

Fig. S3: PCA on microsatellites genotypes in (A) pool frogs (circles) and kleptons (triangles), as well as (B) marsh frog haplotypes phased from kleptons. Colors correspond to the lineages identified from the phylogeny (Fig. 2). In N-Italy, the microsatellites could not disentangle between the admixing *P. lessonae* and *P.* n. t. 2. In (A), the two axes contributed 5.8% and 3.9% of the total variance. Kleptons from S-Italy (*P.* kl. *hispanicus*) have a disruptive clustering on axis 1 compared to *P.* kl. *esculentus*, indicating a different marsh frog ancestor. Moreover, given the different patterns of genetic structure between *P. bergeri* and *P.* kl. *hispanicus*, the marsh frog lineage *P.* n. t. 1 likely diverged prior to hybridogenesis. In (B), the two axes contributed 24.2% and 13.8% of the total variance. The phased *P. ridibundus* and *P.* n. t. 1 haplotypes show distinct clustering, also confirming their different identities. However, some haplotypes from the northern Apennines share similarities that may result from ancient admixture.

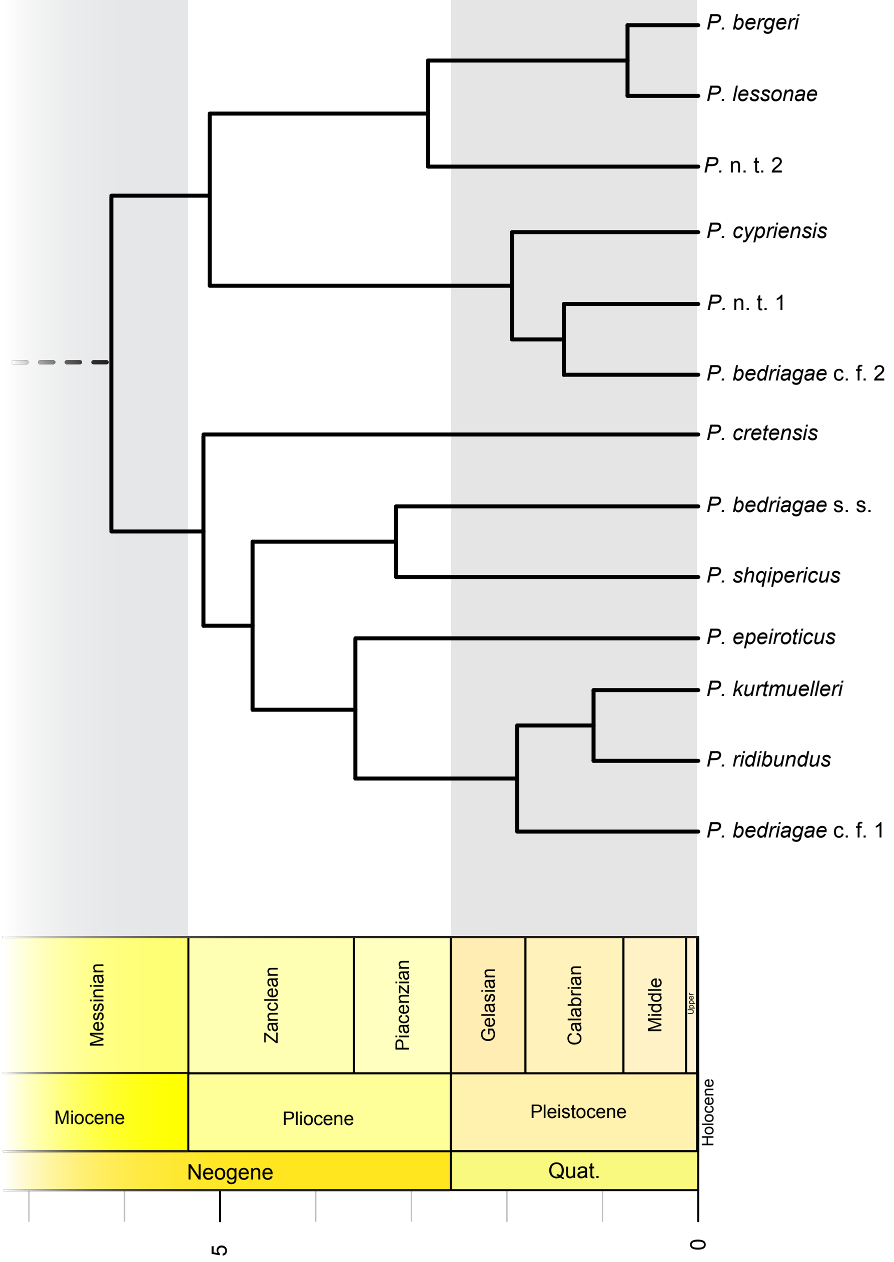

Fig. S4: Time-calibrated phylogeny based on the BEAST runs (in My)

Fig. S5: Location of SAI-1 primers. *P. lessonae / bergeri* - specific primers are in red and primers specific to the other species are in blue. Dash line: *P. lessonae / bergeri* - deletion

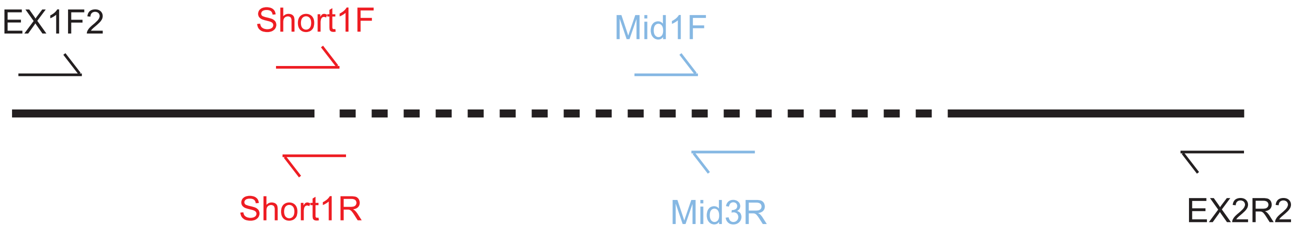

Table S1: Samples used in this study with their taxonomic nature (determined genetically for the study area), mitotypes and nuclear genotypes. Kleptons marked by an asterisk (*P.* kl. *esculentus*) may deserve reconsideration given the nature of their pool frog genome. *Pelophylax* n. t. 1 and n. t. 2 alleles were labelled EXT and TIC, respectively, as a reference to "Extinct" and "Ticino" (where *P.* n. t. 2 was massively found).

ID	Reference	Lat.	Long.	Taxon	mtDNA	SAI-1	CMI-2	STR
Agra1	this study	46.03	8.90	*P.* kl *esculentus**	*P. lessonae*	TIC03 / RID06	TIC05 / NA	×
Agra2	this study	46.03	8.90	*P.* kl *esculentus**	*P. lessonae*	-	-	×
Agra3	this study	46.03	8.90	*P.* kl *esculentus**	-	TIC01 / RID06	LES02 / RID05	
Assunta3	this study	44.43	8.04	*P. lessonae* × *P. bergeri*	*P. bergeri*	LES04 / LES04	BER02 / BER02	×
Assunta1	this study	44.43	8.04	*P. bergeri*	*P. bergeri*	-	BER02 / BER02	×
Cesine5	this study	40.37	18.33	*P. bergeri*	*P. bergeri*	BER04 / BER06	BER02 / BER02	×
Cesine6	this study	40.37	18.33	*P. bergeri*	*P. bergeri*	BER07 / BER07	BER03 / BER03	
Dosolo24	this study	44.95	10.64	*P.* kl *esculentus**	-	LES01 / RID06	TIC05 / RID06	×
Dosolo26	this study	44.95	10.64	*P.* kl *esculentus**	*P. lessonae*	TIC06 / RID06	TIC05 / RID05	
Firenze20	this study	43.77	11.26	*P. bergeri*	*P. bergeri*	-	BER02 / BER02	×
Firenze21	this study	43.77	11.26	*P. bergeri*	*P. bergeri*	BER03 / BER07	BER02 / BER02	×
Firenze22	this study	43.77	11.26	*P. bergeri*	*P. bergeri*	BER07 / BER07	BER02 / BER02	×
Firenze23	this study	43.77	11.26	*P. bergeri*	*P. bergeri*	-	BER02 / BER02	×
Firenze24	this study	43.77	11.26	*P. bergeri*	*P. bergeri*	-	BER02 / BER02	×
Firenze25	this study	43.77	11.26	*P. bergeri*	*P. bergeri*	-	BER02 / BER02	×
Firenze26	this study	43.77	11.26	*P. bergeri*	*P. bergeri*	-	BER02 / BER02	×
Firenze27	this study	43.77	11.26	*P. bergeri*	*P. bergeri*	-	-	×
Firenze28	this study	43.77	11.26	*P. bergeri*	*P. bergeri*	-	BER02 / BER02	×
Firenze29	this study	43.77	11.26	*P. bergeri*	*P. bergeri*	-	BER02 / BER02	×
Firenze30	this study	43.77	11.26	*P. bergeri*	*P. bergeri*	-	BER02 / BER02	×
FT12	this study	41.55	14.88	*P.* kl *hispanicus*	*P. bergeri*	- / EXT01	BER02 / EXT01	×
FT13	this study	41.55	14.88	*P. bergeri*	*P. bergeri*	BER07 / BER07	BER02 / BER02	×
Godelino2	this study	46.17	8.75	*P.* kl *esculentus**	*P. lessonae*	TIC05 / RID03	TIC01 / RID05	×
Godelino3	this study	46.17	8.75	*P.* kl *esculentus**	*P. lessonae*	TIC05 / RID06	TIC01 / RID07	×
Godelino4	this study	46.17	8.75	*P.* n. t. 2	*P. lessonae*	TIC04 / TIC05	TIC01 / TIC01	×
Godelino5	this study	46.17	8.75	*P. lessonae* × *P.* n. t. 2	*P. lessonae*	LES03 / TIC05	LES02 / LES02	×
Godelino6	this study	46.17	8.75	*P.* kl *esculentus**	-	-	-	×

Godelino7	this study	46.17	8.75	*P.* kl *esculentus**	*P. lessonae*	LES02 / RID06	TIC04 / RID07	×
Godelino8	this study	46.17	8.75	*P.* kl *esculentus**	*P. lessonae*	TIC05 / RID06	TIC05 / RID05	×
Godelino9	this study	46.17	8.75	*P. lessonae / P.* n. t. 2	-	-	-	×
Gudo1	this study	46.17	8.95	*P. lessonae x P.* n. t. 2	*P. lessonae*	LES03 / TIC04	LES02 / LES02	×
Gudo2	this study	46.17	8.95	*P.* kl *esculentus**	*P. lessonae*	TIC05 / RID06	TIC05 / -	×
Gudo3	this study	46.17	8.95	*P. lessonae* x *P.* n. t. 2	*P. lessonae*	-	LES02 / TIC01	×
Macerata3	this study	43.30	13.46	*P. bergeri*	*P. bergeri*	BER07 / BER07	BER02 / BER02	×
Macerata5	this study	43.30	13.46	*P. bergeri*	*P. bergeri*	-	BER02 / BER02	×
MNHGE23	this study	46.16	8.78	*P. lessonae / P.* n. t. 2	*P. lessonae*	-	-	×
Noto6	this study	36.89	15.07	*P.* kl *hispanicus*	*P. bergeri*	BER02 / EXT01	BER01 / EXT01	×
Noto7	this study	36.89	15.07	*P.* kl *hispanicus*	*P. bergeri*	-	-	×
Noto10	this study	36.89	15.07	*P.* kl *hispanicus*	*P. bergeri*	-	-	×
Noto11	this study	36.89	15.07	*P.* kl *hispanicus*	*P. bergeri*	-	-	×
Noto12	this study	36.89	15.07	*P.* kl *hispanicus*	*P. bergeri*	-	-	×
Noto13	this study	36.89	15.07	*P.* kl *hispanicus*	*P. bergeri*	-	-	×
Noto14	this study	36.89	15.07	*P.* kl *hispanicus*	*P. bergeri*	-	-	×
Novara10	this study	45.45	8.62	*P.* n. t. 2	*P. lessonae*	TIC07 / TIC08	TIC05 / TIC05	×
Novara6	this study	45.45	8.62	*P.* n. t. 2	*P. lessonae*	TIC09 / TIC10	TIC05 / TIC05	
PAD9	this study	45.41	11.88	*P.* kl *esculentus**	*P. lessonae*	-	-	×
PAD11	this study	45.41	11.88	*P.* kl *esculentus**	*P. lessonae*	-	-	×
Parone1	this study	46.14	8.82	*P. lessonae* x *P.* n. t. 2	*P. lessonae*	LES03 / TIC05	TIC01 / TIC02	×
Parone2	this study	46.14	8.82	*P. lessonae* x *P.* n. t. 2	*P. lessonae*	LES03 / TIC05	TIC05 / TIC05	×
Parone3	this study	46.14	8.82	*P. lessonae* x *P.* n. t. 2	*P. lessonae*	LES01 / TIC09	TIC05 / TIC05	×
Parone4	this study	46.14	8.82	*P.* kl *esculentus** / *P.* n. t. 2 ?	*P. lessonae*	TIC02 / TIC04	TIC05 / RID05	×
Parone5	this study	46.14	8.82	*P. lessonae x P.* n. t. 2	*P. lessonae*	TIC04 / -	LES02 / TIC03	×
Parone6	this study	46.14	8.82	*P.* n. t. 2	*P. lessonae*	TIC07 / TIC08	TIC05 / TIC05	×
Parone7	this study	46.14	8.82	*P. bergeri* x *P.* n. t. 2	*P. lessonae*	TIC05 / TIC05	BER02 / BER02	×
PEBE1	this study	41.84	9.32	*P. bergeri*	*P. bergeri*	BER02 / BER02	BER02 / BER05	×
PEBE2	this study	41.84	9.32	*P. bergeri*	*P. bergeri*	-	BER05 / BER05	×
PEBE3	this study	41.84	9.32	*P. bergeri*	*P. bergeri*	BER07 / BER07	BER05 / BER05	×
PEBE4	this study	41.84	9.32	*P. bergeri*	*P. bergeri*	-	BER05 / BER05	×
PEBE5	this study	41.84	9.32	*P. bergeri*	*P. bergeri*	BER06 / BER06	BER05 / BER05	×
PEBE6	this study	41.84	9.32	*P. bergeri*	*P. bergeri*	-	BER05 / BER05	×
PEBE7	this study	41.84	9.32	*P. bergeri*	*P. bergeri*	-	BER05 / BER05	×

PUA49	this study	44.51	12.22	*P.* n. t. 2	*P. lessonae*	TIC11 / TIC12	TIC01 / TIC02	×
PUA50	this study	44.51	12.22	*P. bergeri* x *P.* n. t. 2	*P. lessonae*	BER07 / TIC03	BER02 / TIC05	×
SC10	this study	40.39	15.59	*P.* kl *hispanicus*	*P. bergeri*	-	-	×
SC11	this study	40.39	15.59	*P. bergeri*	*P. bergeri*	BER01 / BER08	BER02 / BER02	×
TCA20	this study	41.49	12.60	*P. bergeri*	*P. bergeri*	-	BER02 / BER02	×
TCA21	this study	41.49	12.60	*P. bergeri*	*P. bergeri*	BER07 / BER07	BER02 / BER05	×
TF1	this study	43.08	11.26	*P. bergeri*	*P. bergeri*	BER07 / BER07	BER02 / BER02	×
TF2	this study	43.08	11.26	*P. bergeri*	*P. bergeri*	-	BER02 / BER02	×
Vico1bF	this study	42.32	12.17	*P.* kl *hispanicus*	*P. bergeri*	- / EXT01	BER02 / -	×
Vico3bf	this study	42.32	12.17	*P.* kl *hispanicus*	*P. bergeri*	BER06 / -	-	×
Vieste3	this study	41.88	16.17	*P. bergeri*	*P. bergeri*	-	-	×
Vieste4	this study	41.88	16.17	*P. bergeri*	*P. bergeri*	BER04 / BER05	BER02 / BER02	×
VOT12	this study	39.28	16.55	*P.* kl *hispanicus*	*P. bergeri*	BER04 / EXT01	BER01 / EXT01	×
VOT14	this study	39.28	16.55	*P.* kl *hispanicus*	*P. bergeri*	BER04 / EXT01	BER01 / EXT02	
Zomaro8c	this study	38.31	16.11	*P.* kl *hispanicus*	*P. bergeri*	BER04 / EXT01	BER01 / EXT03	×
Zomaro12c	this study	38.31	16.11	*P.* kl *hispanicus*	*P. bergeri*	-	-	x
GM01.2015	this study	42.35	19.04	*P. shqipericus*	-	SHQ01 / SHQ01	SHQ01 / SHQ01	
GM157	this study	34.92	32.90	*P. cypriensis*	-	CYP01 / CYP02	CYP01 / CYP01	
GM.J.01.56 / HE858239	this study / 18	32.20	35.89	*P. bedriagae* s.s.	-	BED01 / BED01	BED01 / BED01	
GM176	this study	36.11	36.25	*P. bedriagae* c.f. 1	-	-	BEDcf1.01 / BEDcf1.02	
GM673	this study	37.23	27.60	*P. bedriagae* c.f. 2	-	-	BEDcf2.01 / BEDcf2.01	
GM79	this study	38.16	20.38	*P. epeiroticus*	-	-	EPE01 / EPE01	
GM213	this study	-	-	*P. cretensis*	-	-	CRE01 / CRE01	
GM051	this study	42.11	27.11	*P. ridibundus*	-	-	RID04 / RID04	
GM206	this study	52.04	29.16	*P. ridibundus*	-	-	RID02 / RID04	
GM240	this study	50.37	26.14	*P. ridibundus*	-	-	RID03 / RID03	
Ronda1	11	36.75	-5.16	*P. perezi*	-	-	PER01 / PER01	
PK1m	11	41.26	19.86	*P. kurtmuelleri*	-	-	KUR01 / KUR01	
PK3	11	41.26	19.86	*P. kurtmuelleri*	-	KUR04 / KUR04	KUR01 / KUR02	
PK4f	11	41.26	19.86	*P. kurtmuelleri*	-	-	KUR01 / KUR01	
PK5f	11	41.26	19.86	*P. kurtmuelleri*	-	KUR01 / KUR03	KUR01 / KUR01	
Grza	this study	43.63	20.24	*P. ridibundus*	-	-	RID01 / RID01	
MF094366	16	-	-	*P. lessonae*	-	LES01	-	

MF094356	16	-	-	*P. lessonae*	-	-	LES01
MF094357	16	-	-	*P. lessonae*	-	-	LES02
HE858212	18	38.37	23.08	*P. ridibundus*	-	RID04 / RID04	-
HE858214	18	37.88	21.29	*P. kurtmuelleri*	-	KUR05 / KUR05	-
HE858215	18	37.81	20.86	*P. kurtmuelleri*	-	KUR07 / KUR07	-
HE858216	18	38.03	21.36	*P. kurtmuelleri*	-	KUR02 / KUR07	-
HE858217	18	38.67	23.07	*P. kurtmuelleri*	-	KUR02 / KUR07	-
HE858218	18	31.55	35.74	*P. bedriagae* s. s.	-	BED02 / BED02	-
HE858220	18	31.55	35.74	*P. bedriagae* s. s.	-	BED01 / BED01	-
HE858221	18	32.62	35.64	*P. bedriagae* s. s.	-	BED02 / BED02	-
HE858223	18	36.50	36.45	*P. bedriagae* c. f. 1	-	BEDcf1.01 / BEDcf1.01	-
HE858224	18	37.28	37.12	*P. bedriagae* c. f. 1	-	BEDcf1.01 / BEDcf1.01	-
HE858225	18	36.69	35.63	*P. bedriagae* c. f. 1	-	BEDcf1.01 / BEDcf1.01	-
HE858226	18	36.69	35.63	*P. bedriagae* c. f. 1	-	BEDcf1.01 / BEDcf1.01	-
HE858227	18	35.74	27.17	*P. bedriagae* c. f. 2	-	BEDcf2.02 / BEDcf2.02	-
HE858228	18	36.31	28.14	*P. bedriagae* c. f. 2	-	BEDcf2.02 / BEDcf2.02	-
HE858230	18	37.21	30.94	*P. bedriagae* c. f. 2	-	BEDcf2.03 / BEDcf2.03	-
HE858231	18	37.68	31.72	*P. bedriagae* c. f. 2	-	BEDcf2.01 / BEDcf2.06	-
HE858232	18	39.61	27.02	*P. bedriagae* c. f. 2	-	BEDcf2.05 / BEDcf2.05	-
HE858233	18	38.67	26.76	*P. bedriagae* c. f. 2	-	BEDcf2.05 / BEDcf2.07	-
HE858235	18	38.61	27.45	*P. bedriagae* c. f. 2	-	BEDcf2.04 / BEDcf2.08	-
HE858237	18	36.31	33.96	*P. bedriagae* c. f. 2	-	BEDcf2.03 / BEDcf2.03	-
HE858238	18	37.23	29.71	*P. bedriagae* c. f. 2	-	BEDcf2.01 / BEDcf2.01	-
HE858240	18	31.45	35.79	*P. bedriagae* s. s.	-	BED01 / BED01	-
FN432363	17	47.54	52.39	*P. ridibundus*	-	RID01 / RID02	-
FN432364	17	52.41	14.54	*P. ridibundus*	-	RID05 / RID05	-
FN432365	17	52.38	16.67	*P. ridibundus*	-	RID06 / RID06	-
FN432366	17	37.88	21.29	*P. kurtmuelleri*	-	KUR06 / KUR06	-
FN432367	17	38.03	21.36	*P. kurtmuelleri*	-	KUR07 / KUR07	-
FN432368	17	32.67	36.73	*P. bedriagae* s. s.	-	BED03 / BED03	-
FN432370	17	37.92	21.27	*P. epeiroticus*	-	EPE01 / EPE01	-
FN432372	17	38.36	26.14	*P. bedriagae* c. f. 2	-	BEDcf2.04 / BEDcf2.09	-
FN432373	17	37.11	28.47	*P. bedriagae* c. f. 2	-	BEDcf2.05 / BEDcf2.10	-
FN432376	17	35.06	25.31	*P. cretensis*	-	CRE01 / CRE01	-

FN432377	17	43.53	4.75	*P. perezi*	-	PER01 / PER01	-
FN432381	17	40.37	16.80	*P. bergeri*	-	BER07 / BER07	-
FN432382	17	39.62	16.27	*P. bergeri*	-	BER06 / BER06	-
FN432384	17	52.67	13.35	*P. lessonae*	-	LES01 / LES01	-
FN432385	17	52.57	12.92	*P. lessonae*	-	LES01 / LES01	-
FN432386	17	-	-	*P. nigromaculata*	-	NIG01 / NIG01	
AB980345-46	35	36.99	35.33	*P. ridibundus*	-	-	RID02 / BEDcf1.01
AB980347-48	35	-	-	*P. lessonae*	-	-	LES02 / LES02
AB980349-50	35	33.22	132.56	*P. nigromaculata*	-	-	NIG01 / NIG01
AB980295-96	35	43.77	11.26	*P. bergeri*	-	-	BER02 / BER04

Table S2: Primers used in this study. PCRs were carried out in 25µL reaction, with 7.5µL of Qiagen MultiPlex Master Mix (MPMM), 1µL of each primer (10µL) and 3µL of DNA template (5-30 ng.µL^{-1}). Conditions were as follow: 15’ at 95°C, 38 cycles of 30” at 94°C, 45” at 55°C and 1’ at 72°C, and 10’ at 72°C.

Marker	Primer	Sequence (5'-3')	Reference
SAI-1	EX1F2	ACTCTGATTTGTCTGTTTATTC	17
	EX2R2	CTGCCTTTACAATATCGTTTAT	17
	Mid1F	AACTTTTCAGAGAAAGGGAA	this study
	Mid3R	TAAAGAACCCCTTAYGCAGT	this study
	Short1F	ATATCGGTACGGTACGGTTA	this study
	Short1R	TAACCGTACCGTACCGATAT	this study
CMI-2	MYELF1	CAGTGAATGACAGCATTTCCAG	16
	MYELR3	GTCAAAGCCTTCAAAGACCATTG	16

Table S3: GenBank accession numbers of haplotypes.

Marker	Haplotype	GenBank #
SAI-1	LES01	MF667593
SAI-1	LES02	MF667594
SAI-1	LES03	MF667595
SAI-1	LES04	MF667596
SAI-1	BER08	MF667598
SAI-1	BER02	MF667599
SAI-1	BER03	MF667600
SAI-1	BER04	MF667601
SAI-1	BER01	MF667602
SAI-1	BER05	MF667603
SAI-1	BER06	MF667604
SAI-1	BER07	MF667605
SAI-1	TIC01	MF667606
SAI-1	TIC02	MF667607
SAI-1	TIC03	MF667608
SAI-1	TIC09	MF667609
SAI-1	TIC04	MF667610
SAI-1	TIC05	MF667611
SAI-1	TIC06	MF667612
SAI-1	TIC10	MF667613
SAI-1	TIC07	MF667614
SAI-1	TIC11	MF667615
SAI-1	TIC12	MF667616
SAI-1	TIC08	MF667617
SAI-1	BEDcf2.01	MF667618
SAI-1	BEDcf2.06	MF667619
SAI-1	BEDcf2.02	MF667620
SAI-1	BEDcf2.03	MF667621
SAI-1	BEDcf2.04	MF667622
SAI-1	BEDcf2.09	MF667623
SAI-1	BEDcf2.07	MF667624
SAI-1	BEDcf2.10	MF667625
SAI-1	BEDcf2.05	MF667626
SAI-1	BEDcf2.08	MF667627
SAI-1	CRE01	MF667628
SAI-1	EPE01	MF667629
SAI-1	BED01	MF667630
SAI-1	BED02	MF667631
SAI-1	BED03	MF667632
SAI-1	BEDcf1.01	MF667633

SAI-1	KUR03	MF667634
SAI-1	KUR01	MF667635
SAI-1	KUR02	MF667636
SAI-1	KUR06	MF667637
SAI-1	KUR07	MF667638
SAI-1	KUR05	MF667639
SAI-1	KUR04	MF667640
SAI-1	RID01	MF667641
SAI-1	RID02	MF667642
SAI-1	RID03	MF667643
SAI-1	RID04	MF667644
SAI-1	RID05	MF667645
SAI-1	RID06	MF667646
SAI-1	EXT01	MF667647
SAI-1	SHQ01	MF667648
SAI-1	CYP01	MF667649
SAI-1	CYP02	MF667650
SAI-1	PER01	MF667651
SAI-1	NIG	MF667653
CMI-2	CYP01	MF667654
CMI-2	EPE01	MF667655
CMI-2	SHQ01	MF667656
CMI-2	BEDcf1.02	MF667657
CMI-2	BEDcf1.01	MF667658
CMI-2	BED01	MF667659
CMI-2	BEDcf2.01	MF667660
CMI-2	PER01	MF667661
CMI-2	CRE01	MF667662
CMI-2	KUR02	MF667663
CMI-2	KUR01	MF667664
CMI-2	RID01	MF667665
CMI-2	RID02	MF667666
CMI-2	RID03	MF667667
CMI-2	RID04	MF667668
CMI-2	RID05	MF667669
CMI-2	RID06	MF667670
CMI-2	RID07	MF667671
CMI-2	RID08	MF667672
CMI-2	LES01	MF667673
CMI-2	LES02	MF667674
CMI-2	BER01	MF667675
CMI-2	BER02	MF667676
CMI-2	BER03	MF667677
CMI-2	BER04	MF667678

CMI-2	BER05	MF667679
CMI-2	TIC01	MF667680
CMI-2	TIC02	MF667681
CMI-2	TIC03	MF667682
CMI-2	TIC04	MF667683
CMI-2	TIC05	MF667684
CMI-2	EXT02	MF667685
CMI-2	EXT01	MF667686
CMI-2	EXT03	MF667687
CMI-2	NIG01	MF667688
